# Vascular reconstruction of the decellularized biomatrix for whole-organ engineering—a critical perspective and future strategies

**DOI:** 10.3389/fbioe.2023.1221159

**Published:** 2023-11-13

**Authors:** Santosh Gupta, Akriti Sharma, Goran Petrovski, Rama Shanker Verma

**Affiliations:** ^1^ Stem Cell and Molecular Biology, Laboratory, Department of Biotechnology, Bhupat and Jyoti Mehta School of Biosciences. Indian Institute of Technology Madras, Chennai, India; ^2^ Center for Eye Research and Innovative Diagnostics, Department of Ophthalmology, Institute for Clinical Medicine, Faculty of Medicine, University of Oslo, Oslo, Norway; ^3^ Center for Eye Research and Innovative Diagnostics, Department of Ophthalmology, Institute for Clinical Medicine, University of Oslo, Oslo, Norway; ^4^ Department of Ophthalmology, Oslo University Hospital, Oslo, Norway; ^5^ Department of Ophthalmology, University of Split School of Medicine and University Hospital Centre, Split, Croatia

**Keywords:** vascular reconstruction, artificial organ, whole-organ engineering, decellularized organ, endothelialization

## Abstract

Whole-organ re-engineering is the most challenging goal yet to be achieved in tissue engineering and regenerative medicine. One essential factor in any transplantable and functional tissue engineering is fabricating a perfusable vascular network with macro- and micro-sized blood vessels. Whole-organ development has become more practical with the use of the decellularized organ biomatrix (DOB) as it provides a native biochemical and structural framework for a particular organ. However, reconstructing vasculature and re-endothelialization in the DOB is a highly challenging task and has not been achieved for constructing a clinically transplantable vascularized organ with an efficient perfusable capability. Here, we critically and articulately emphasized factors that have been studied for the vascular reconstruction in the DOB. Furthermore, we highlighted the factors used for vasculature development studies in general and their application in whole-organ vascular reconstruction. We also analyzed in detail the strategies explored so far for vascular reconstruction and angiogenesis in the DOB for functional and perfusable vasculature development. Finally, we discussed some of the crucial factors that have been largely ignored in the vascular reconstruction of the DOB and the future directions that should be addressed systematically.

## 1 Introduction

Whole-organ re-engineering is one of the few available options in the field of tissue engineering and regenerative medicine, bridging the gap between patients requiring organs and availability of the organ (Badylak et al., 2011; Jungebluth and Macchiarini, 2011; Badylak et al., 2012). There are always long waiting lists of patients requiring organ transplant, but due to a shortage of suitable organs, many patients cannot undergo organ transplants, resulting in mortality. The primary reasons for the unsuitability of an organ are the mismatch of an organ for transplantation for a given patient, transportation, graft rejection observed over a period of time, graft underperformance upon transplantation, and long-term dependence on immunosuppressive drugs by the patients upon graft transplantation (Welman et al., 2015). Such processes lead to compromise in the patient’s compliance. Therefore, the technically acceptable solution is to create an artificial organ using a biologically relevant whole-organ biomatrix and the patient’s derived parenchymal and non-parenchymal cells that constitute a given organ (Pellegata et al., 2018).

Such approaches can be well addressed by pluripotent stem cells (PSCs) such as induced pluripotent stem cells (iPSCs), which can be used to derive any cell type present in the human body. PSCs have been shown to differentiate into functional cells such as hepatocytes, cardiomyocytes, endothelial cells, pericytes, mesenchymal stem cells (MSCs), smooth muscle cells (SMCs), myocytes, and macrophages (Peloso et al., 2015a). Such cells that play an essential role in the physiological function of a given organ, in principle, can be used to reconstruct an artificial organ by seeding them in a suitable scaffold (Ott et al., 2008; Uygun et al., 2010a). In this regard, the most convenient approach that closely resembles the native architecture of the organ can be derived using organ decellularization technology. Such a method provides a native biomimetic scaffold with all the existing extracellular components present in a given native organ, along with the existing blood vessel, making the whole organ perfusable the way it does under *in vivo* conditions (Baptista et al., 2009; Moran et al., 2014). Therefore, recellularizing such a decellularized scaffold with a set of parenchymal and non-parenchymal cells can be a practical guide to fabricate a functional, transplantable organ (Tapias and Ott, 2014; Peloso et al., 2015b; Sohn et al., 2020).

Ever since the first report of the decellularized biomatrix for reconstructing the whole organ was published in 2008 (Ott et al., 2008), almost all major vital organs, such as the heart, liver, kidneys, pancreas, lungs, and intestine, have been studied for *in vitro* artificial organ development. However, a major limitation with such an organ development strategy is to recreate the blood vessel by utilizing the pre-existing blood vessel structure (Hussein et al., 2020). The first study to report re-endothelialization of existing blood vessels was published in 2011. The authors injected human umbilical vein endothelial cells (HUVECs) into the portal vein of the hepatocyte-infused liver to study revascularization of the scaffold. Similarly, many other organs such as the heart (Ott et al., 2008), liver (Uygun et al., 2010a), lungs (Ott et al., 2010), and pancreas have been studied. However, such approaches have been straightforward and include a simple injection of endothelial cells into the existing blood vessels and relying on the ability of the endothelial cells to attach to the existing blood vessel at a macro-level. However, no focused approach has been proposed to target or control micro-vessel formation in those studies (Ott et al., 2010; Rouwkema and Khademhosseini, 2016; Pellegata et al., 2018).

Here, we discussed the strategies explored so far for vascular reconstruction in decellularized organs. We also focused on various strategies that either have not been attempted or require attention in a multi-parametric approach to recreating the vasculature on the pre-existing vascular structures and, at the same time, controlling the formation of micro-vessels. Eventually, we outlined the future direction of re-endothelialization of decellularized organs and the areas requiring attention to achieve a vascularized functional, transplantable organ for clinical use.

## 2 Why is endothelialization essential in whole-organ re-engineering?

One major limitation in the successful clinical translation of large-scale tissue-engineered organs is vascularization (Hussein et al., 2020; Rouwkema and Khademhosseini, 2016). The formation of both macroscale vessels (arteries and arterioles) and micro-vessels (capillaries) in a complex scaffold of specific topology and microarchitecture with a defined biochemical composition is significantly challenging (Song et al., 2018; Yang et al., 2020). Another reason why it is still challenging to recreate the functional vasculature in a decellularized organ biomatrix is that it mimics native vascular structures in that particular organ.

Physiologically, the importance of vascularization has been elucidated in detail previously and is not covered extensively in this article. The primary importance of functional vasculature from a tissue-engineered construct is the perfusion of the construct with the host blood for the delivery of nutrients, gaseous exchange, growth factors (GFs), and maintenance of the homeostatic balance essential for the function and survival of the construct. Its absence leads to necrosis-induced death and underperformance of the graft, followed by rejection of the transplanted graft (Song et al., 2018; Chandra and Atala, 2019).

Few reports exist where the authors have studied the blood perfusion capability of the decellularized organ biomatrix without re-endothelialization, both *ex vivo* and *in vivo*. The primary target of such studies was to decipher the effect of blood reperfusion on coagulation, its fate, and the vascular perfusion capability as a function of time in the decellularized organ biomatrix, especially when it is transplanted *in vivo* (Orlando et al., 2012; Bao et al., 2015). One of the significant problems with decellularized organs for clinical transplantation is their thrombogenicity (Uygun et al., 2012). Orlando et al. (2012) showed that a decellularized porcine kidney, when transplanted *in vivo*, can easily be reperfused and sustained blood pressure and well tolerated. Upon implantation, satisfactory blood flow throughout the whole acellular renal scaffold could be observed for 60 min. However, after a 2-week follow-up, it was observed that a massive non-specific inflammatory infiltrate, which was limited to the pericapsular zone and scaffold parenchyma, was scattered with rare inflammatory cells. The vascular tree appeared to be completely obstructed by thrombi and trapped red blood cells. This highlights the first rationality for endothelialization of the decellularized organ and the absence of re-endothelialized organ-associated reactions upon *in vivo* implantation. Since the major reason for coagulation or thrombosis is the exposure of circulating platelets in the blood to collagen of the decellularized matrix (Ott et al., 2008; Uygun et al., 2010b), this becomes critical for the complete endothelialization of the decellularized matrix for transplantation and possible clinical application. Therefore, related studies can be considered a base to understanding why endothelialization is essential for the normal function and acceptance of the graft for long-term sustainability. On the contrary, when similar studies were conducted culturing ECs in the decellularized organ biomatrix, better acceptability with minimal coagulation and improved physiological function could be observed. Furthermore, platelet activation could be minimized significantly, and reduced calcification was observed compared to the non-endothelialized decellularized organ biomatrix (Shirakigawa et al., 2012; Kojima et al., 2018). Kojima et al. (2018) used rat liver sinusoidal endothelial cells (LSECs), along with hepatocytes, to repopulate and re-endothelialize the rat decellularized whole liver. They found that re-endothelialization improved the hepatocyte functions as well as significantly reduced thrombogenicity compared to non-endothelialized decellularized organs with extracorporeal blood perfusion. This study highlights the importance of endothelialization to improve the graft function while reducing thrombogenicity.

In practice, it is difficult to achieve complete endothelialization of a decellularized organ. This could be observed in various studies where endothelialized decellularized organs, when administered with anticoagulants, led to decreased thrombogenicity but showed complications such as hemorrhage and failure of the recipients to survive more than 8 h. For such reasons, most *in vivo* transplantation studies have been limited to less than 24 h (Uygun et al., 2010b; Bao et al., 2011; Barakat et al., 2012). Therefore, new approaches and innovations have been investigated to improve endothelialization and reduce the undesirable physiological outcome. For example, coating of the decellularized matrix with an anticoagulant such as heparin (Hussein et al., 2016; Bao et al., 2015) or other materials such as gelatin (Tang-Quan et al., 2020) could promote endothelialization. Furthermore, the efficiency has been improved by modifying the decellularized matrix with binding moieties such as antibodies (Ko et al., 2014) and peptides (Jiang et al., 2016), promoting EC attachment. Hence, such studies underline the importance of endothelialization of the DOB for *in vivo* optimal perfusion and function of artificial organs.

## 3 Factors studied for vascular reconstruction of the DOB

**FIGURE 1:**
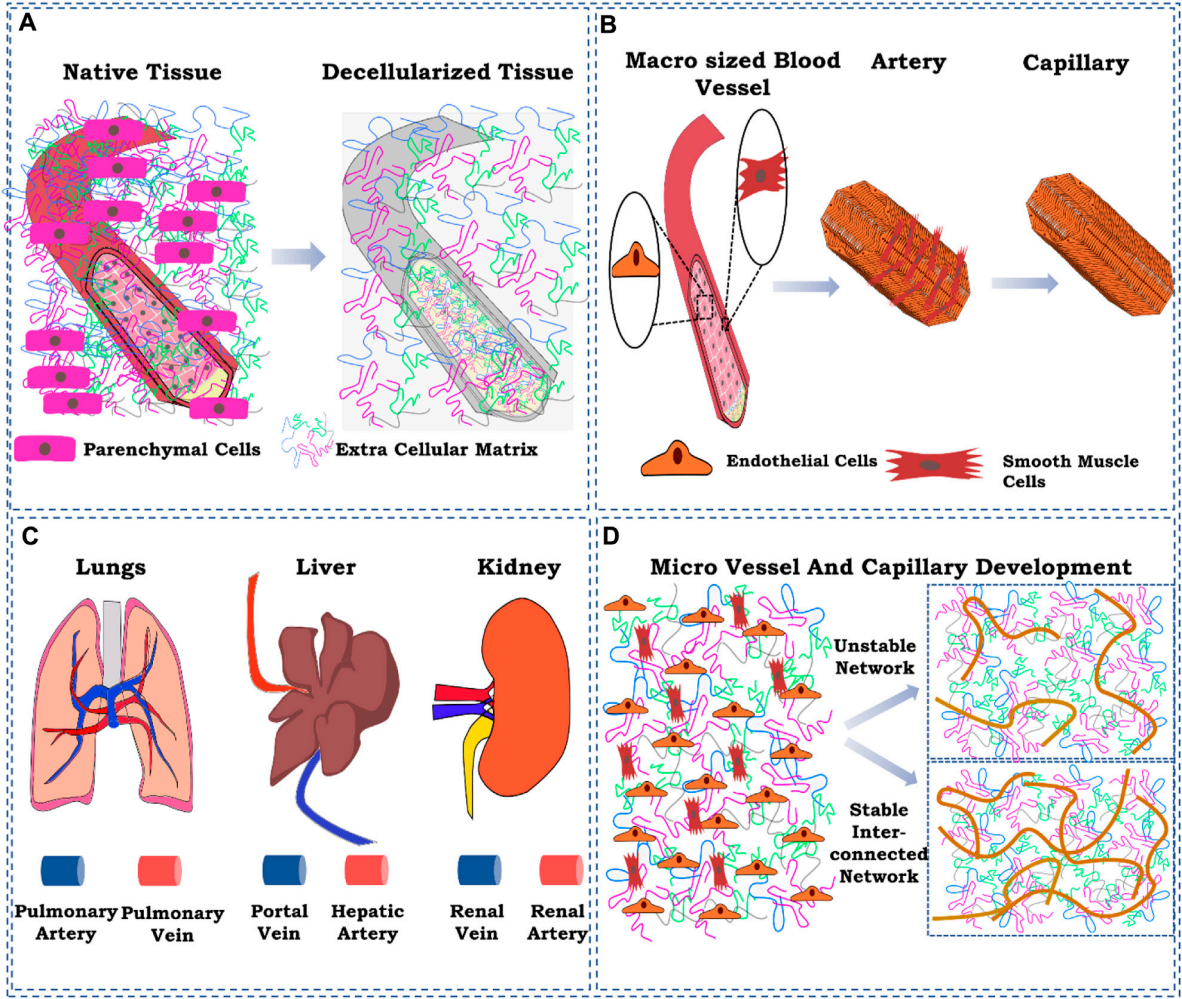
Schematic representation of the ignored factors while reconstructing the vasculature in the decellularized whole-organ matrix. **(A)** ECM component loss of the existing blood vessel as a result of decellularization; **(B)** cellular composition of a blood vessel at the macro-and micro-levels; **(C)** delivery route for cells for vascular reconstruction; **(D)** controlling spatiotemporal development of micro-vessels and capillaries. The figures were prepared using Inkscape open-source vector graphics editor software, United States

General principles explored so far are the simple injection of endothelial cells using the cannulated blood vessel and then relying on the adherence capability of the cells (Shirakigawa et al., 2012). Recent approaches have been shown to modify the adherence capability of existing blood vessels to endothelial cells by the addition/incorporation of factors that enhance and/or promote the adherence of endothelial cells to the existing blood vessel structures. However, such approaches focus only on the existing patent blood vessel structures. They do not focus significantly on micro-vessel formation (Shirakigawa et al., 2013) and the overall structural stability of the re-engineered blood vessel in terms of its cellular heterogeneity that controls the physiological response of the organ by vasodilation and vasoconstriction, neurological behavior, immune system milieu, and response to body’s various biochemical, biophysical, and physiological responses. Here, we elucidated some essential aspects of re-engineering the vasculature of a decellularized organ biomatrix. These factors have not been extensively studied so far and require detailed and definite impetus while developing strategies for the vascular reconstruction of a decellularized organ biomatrix (Figure 1).

### 3.1 Extracellular matrix component loss of the existing blood vessel

In the decellularized organ biomatrix, the patent blood vessel exists. Therefore, creating macro-to-micro-sized vessels with a defined contour and three-dimensional network is unnecessary. However, the matrix composition changes the larger blood vessel structure due to the use of detergent-based decellularization protocols. Although successful in eliminating all the cellular components from a whole organ, such methods also result in the concomitant loss of extracellular matrix (ECM) components (Petersen et al., 2012; Nonaka et al., 2014a). However, such a loss of matrix components has not been characterized. Therefore, it is challenging to identify the loss of extracellular matrix components occurring in the parenchymal space or the blood vessel structures to a certain extent.

Moreover, it has been shown that collagen and elastin alignment plays a significant role in maintaining the mechanical behavior of decellularized cardiac tissue (Merna et al., 2013; Ijima et al., 2018). In another study, lungs decellularized using 3-[(3-cholamidopropyl) dimethylammonio]-1-propanesulfonate (CHAPS) and sodium dodecyl sulfate (SDS) were studied for the loss of extracellular matrix materials. It was observed that using CHAPS prevented collagen loss, which was approximately 80% when SDS was used for decellularization. In addition, CHAPS-based decellularization showed better tensile strength and elastic behavior, which were significantly decreased in the SDS group, thus emphasizing the choice and decellularization protocol selection (Petersen et al., 2012). The ionic and non-ionic detergents used for such strategies lead to loss of the ECM components and eventually affect cellular behavior at the macro- and micro-levels.

Therefore, the re-endothelialization strategy for the decellularized matrix should also consider the loss of extracellular matrix components from the vascular structures and the influence of the loss of those materials on re-endothelialization of patent vascular structures. However, specific strategies have been explored to optimize the loss of mechanical property in decellularized organs. For example, Urbano et al. (2017) used two different routes, namely, tracheal and perfusion through blood vessels, to study the change in mechanics of the decellularized lungs of mice. It was observed that there was no significant difference in the mechanical behavior of the decellularized lungs through tracheal or perfusion routes, suggesting that the route would not play a critical role. However, further investigation concerned the organs as the physiology and blood perfusion rates and routes are different for different organs. Another method was studied to see if it influences the mechanical behavior of whole decellularized lungs. Freeze–thawing the organ showed a minor change in the mechanical properties of the organ scaffold compared to the conventional methods of decellularization without preservation in low temperature. This is very significant as the preservation of the retrieved organ is the only way of extending the availability of organs for whole artificial organ engineering (Nonaka et al., 2014b). Subjecting the organ either before decellularization or during decellularization can result in the loss of various ECM components that can influence organ mechanical behavior and cellular function. Therefore, this aspect has to be defined with a standardized protocol in future.

### 3.2 Cellular composition of the blood vessel at the macro- and micro-levels

The hierarchy of blood vessel structures from the artery and vein to an arteriole, venule, and, eventually, to capillaries is supported by several cells that maintain the structure and function of vascular structures and perfusion of the whole organ. The higher complex structures such as artery and vein branches from the descending aorta in the case of major lower and upper abdominal organs are composed of endothelial cells that line the lumen of blood vessels (Devillard and Marquette, 2021). As we move outward from the lumen basement membrane, the layer between endothelial cells and smooth muscle cells regulated various parameters such as constriction and dilation by a series of local and trophic signaling processes. These cells are arranged in two layers forming a perpendicular orientation between the first and second layers of cells. Such an arrangement of cells is a significant determinant of their vasodilation and vasoconstriction activity (Tennant and McGeachie, 1990). Moreover, pericytes and mesenchymal stromal/stem cells are also found to maintain the matrix components of the blood vessels and are responsible for repairing the vessel structure (Tennant and McGeachie, 1990; Song et al., 2018).

### 3.3 Delivery route for cells

The route of cell delivery is critical to consider while strategizing the vascular reconstruction of a given decellularized organ biomatrix. The distribution of seeded cells in the decellularized organ matrix parenchymal space is influenced by the route through which cells are introduced into the decellularized organ. For example, Robertson et al. (2014), to re-create vascular structures in the decellularized heart, introduced endothelial cells in the decellularized organ by three routes: 1) through retrograde aortic infusion; 2) brachiocephalic artery (BA) infusion; and 3) combination of both inferior vena cava plus brachiocephalic artery infusion. Endothelial cell seeding through the inferior vena cava and brachiocephalic artery showed the distribution of cells in the whole decellularized heart. Reseeding through this route not only showed increased proliferation, coverage, and lining of the blood vessel by endothelial cells but also reduced thrombogenicity compared to the other two routes, namely, retrograde aortic infusion and brachiocephalic artery (Robertson et al., 2014). It must also be noted that capillary and micro-vessels in specific organs such as the liver (Braet and Wisse, 2002) and kidney (Satchell and Braet, 2009) are fenestrated with typical fenestration sizes specific to the function of each organ. Therefore, considering this aspect while reconstructing vascular structure in a decellularized organ is critical. This can be included by the route through which endothelial cells are introduced into the decellularized organ matrix for vascular reconstruction. For example, in decellularized kidney vascular reconstruction (Ciampi et al., 2019), iPSC-derived endothelial cells, when introduced through both the renal artery and vein, showed uniform distribution in all compartments, including glomerular capillaries to peritubular capillaries and small vessels. This strategy resulted in the development of fenestration in the glomerular capillaries but not vascular capillaries.

Similarly, Baptista et al. (2011) seeded endothelial cells through the portal vein and vena cava to study the distribution and localization of endothelial cells in the parenchymal and non-parenchymal space. When introduced through the portal vein localized in the periportal region, cells were observed, whereas when seeded through the vena cava, they were localized in the pericentral area). This process underlines the importance and need to study the route of endothelial introduction in the decellularized organ and study the blood vessel lining coverage from the large vessel to capillary level. Each organ is different, so each must be studied separately. The study outcome of one vascularized organ reconstruction should be carefully correlated with that of the other organs, especially when the organs have both tightly controlled leak-proof vessels to fenestrated endothelial lining such as the liver and kidney.

### 3.4 Controlling the spatiotemporal development of micro-vessels and capillaries

The knowledge gained so far regarding the development of micro- and macro-sized blood vessels in the laboratory by mimicking the concepts and principles used during vascularization *in utero* and *in vivo* helped create spatially and temporally controlled vascular structures in microtissue (Cao et al., 2007). However, recreating the blood vessel in a laboratory for whole artificial organ development is an uphill task that the scientific community started over a decade ago. In engineering, any tissue destined for transplantation must have well-developed blood vessels which vary in size and composition, among other regulating factors of vascularization (Cao et al., 2007; Kottamasu and Herman, 2018). In this regard, the proper development of spatiotemporally controlled vascular structures in the decellularized organ matrix becomes highly critical and, at the same time, challenging (Hussein et al., 2020). Many strategies have been used as growth factors and for controlling the mechanical properties, including extracellular matrix components such as glycosaminoglycan at a tissue-level redevelopment. Such strategies need to be recapitulated at the whole-organ level (Kottamasu and Herman, 2018). Very few reports exist where the authors have used growth factor loading to prove vessel development at both capillary and larger-vessel levels. The primary concern relating to vascular reconstruction is forming micro-vessels that do not have a thick and well-established vascular structure observed after decellularization. There is no report studying the fate of the basement membrane of the micro-vessels and capillary structures upon decellularization. It could be possible that due to detergent-based perfusion, the basement membrane becomes dissolved or removed as it is very thin, i.e., approximately 50 nm. So reconstruction of micro-blood-vessels solely depends on endothelial cell ability to proliferate and induce angiogenic and neo-vasculogenic processes in the parenchymal space of the decellularized scaffold. Such processes can be accelerated by adding angiogenic factors and other strategies that promote angiogenic processes when cultured in a bioreactor system in the laboratory.

## 4 Factors used for re-endothelialization/vascular development

Categorizing components for successful re-endothelialization is essential for developing strategies for vascular reconstruction of the decellularized organ biomatrix. The factors can be categorized into cells, pro-angiogenic mitogen factors, small molecules, and microRNA and vector-based gene expression to deliver a quantifiable number of pro-angiogenic factors for the long term (Moon and West, 2008; Rouwkema et al., 2008; Lovett et al., 2009). The selection of endothelial cells for vascular reconstruction in most cases is based on demonstrating proof-of-concept studies. Inducing neovasculogenesis and angiogenesis in the decellularized organ biomatrix is another concern that has been studied by culturing the decellularized organ biomatrix injected with endothelial cells in a bioreactor. Such approaches need to be streamlined to define a well-established and reproducible protocol that can be used to re-create the vasculature in the decellularized organ biomatrix. Here, we discussed, in general, a very narrow focus on the factors that have been potentially used for endothelialization and vascular reconstruction in the decellularized whole-organ biomatrix.

### 4.1 Cell types

A major limitation in designing vascularized whole organs is the selection of endothelial cells. The major problem with primary endothelial cells is their decreased proliferative potential and function when cultured *in vitro*, reducing the possibility of expanding primary cells to produce a high number of cells to fabricate whole organs. However, these cells efficiently perform critical functions such as preventing blood clots by forming a cell barrier between blood vessels and the parenchymal space in an experimental setup. Pooling endothelial cells from multiple tissues is another approach. However, such an approach leads to a heterogeneous population of primary endothelial cells with various proliferative and functional properties, limiting functional vasculature reconstruction upon *in vivo* transplantation (Yang et al., 2020).

#### 4.1.1 Human umbilical vein endothelial cells

HUVECs are the gold standard in vascular biology research, especially in tissue engineering and vascular graft reconstruction research. However, they have certain limitations in terms of their yield and passage *in vitro* during expansion, focusing on developing whole organ vascular reconstruction. In addition, they exhibit poor engraftment and anastomosis upon *in vivo* transplantation in various animal models (Auger et al., 2013). Owing to the extensive use of HUVECs in re-endothelialization studies, the progress in the field of tissue engineering has developed with a particular focus on re-vascularization of the whole decellularized matrix. Ko et al. (2015) showed for the first time that HUVECs could be used for re-vascularization of the human-scale decellularized porcine liver, which, upon *in vivo* transplantation in pigs, could sustain perfusion by the host blood up to 24 h with a significant reduction in platelet adhesion and clot formation. This was achieved by conjugating anti-endothelial cell antibodies to the decellularized liver to improve endothelial cell engraftment and attachment and to maximize the coverage of the vessel wall endothelialization and capillary formation. Similarly, many studies have utilized modification strategies to improve the vascularization of decellularized scaffolds using anti-CD31 aptamers and HUVECs to improve endothelialization of the decellularized liver (Kim et al., 2021). This approach has also showed significant reduction in platelet formation compared to the non-anti-CD31 aptamer-conjugated HUVEC endothelialization, while perfusable blood vessel formation was evident as shown by the connection between the graft and renal circulation obtained. Wan et al. (2020) showed that the decellularized pancreatic organ upon conjugation with GRGDSPC peptides (modified RGD sequence for improved cell attachment) and repopulation with HUVECs showed survival and accelerated the proliferation of HUVECs, with overall angiogenesis being significantly improved compared to the untreated scaffolds. Several studies have utilized HUVECs and chemically modified the decellularized scaffold to show the re-endothelialization of the scaffold, thus, in principle, proving that the re-introduction of endothelial cells such as HUVECs can re-develop the vasculature using already existing patent blood vessel ECM and promoting further capillarization.

#### 4.1.2 Endothelial progenitor cells

Another source of adult endothelial cells is endothelial progenitor cells (EPCs) which can be harvested from blood. These cells are autologous and can be more practically used in the fabrication of vascularized tissue. However, similar limitations also exist regarding their yield and loss of functionality and proliferation when cultured *in vitro* for further expansion (Peters, 2018). Such a source of cells cannot be used in the vascular reconstruction of whole decellularized organs or part of the organ as in the liver. An organ fabrication strategy would not be viable for EPCs as it requires the isolation of a large volume of blood, which is limited by the low yield of such cells from the blood (Finkenzeller et al., 2007). The advantage of EPCs, however, is that by expressing specific cell markers, these circulating cells can attach to the endothelium at specific sites and promote new vessel development (Yoder, 2012). It has also been reported that EPCs have higher angiogenic potential than ECs (Wang et al., 2012). Guo et al. (2018) showed that the decellularized pancreas could be re-endothelialized by EPCs; the result showed that EPCs were located around the blood vessel wall, and the re-endothelialized pancreatic matrix was connected with the host through new blood vessel formation compared to the control group with re-endothelialization. These methods emphasize the importance of any large-scale tissue pre-vascularization for fast host integration. Zhou et al. (2016) used EPCs and hepatocytes to recellularize the decellularized rat liver. Histological examination showed better EPC and hepatocyte growth in the decellularized matrix. *Ex vivo* perfusion of cultures for 3 days showed coverage of the internal surface with the vessel tubular structure. In their study, bone marrow-derived mononuclear cells (BMMNCs) were used to isolate EPCs. The non-adherent cells after 24 h of culture were used in an endothelialization study and confirmed to be positive for CD133 and CD31.

Studies on EPC-based whole decellularized organ vasculature development are limited. Factors, such as the source of EPC isolation, expression of CD133 and CD31 markers, and functionality during *in vitro* culture and yield, remain to be standardized for future re-vascularization studies. However, the aspect of using EPCs has gained significant interest and may be used together with other supporting cell types that promote angiogenesis and vascularization.

#### 4.1.3 MSCs and MSC-derived endothelial cells

Mesenchymal stem cells are a subpopulation of multipotent stem/stromal cells isolated from diverse sources such as bone marrow, adipose tissue, and umbilical cord. These cell types have been differentiated into endothelial-like cells (Tao et al., 2016). We worked on the differentiation of bone marrow-derived MSCs into endothelial-like cells in the decellularized pericardium (Mathapati et al., 2014). Cells were successfully differentiated into endothelial-like cells in the decellularized matrix and showed LDL uptake. In another study, bone marrow-derived MSCs were seeded in the decellularized rat liver. The recellularized organ was cultured for 30 days. The cells showed markers for hepatic sinusoidal endothelial cells (mannose, Fc, and stabilin receptors), along with cell alignment reminiscent of the endothelium (Harper et al., 2020). Alternatively, many mechanisms have been proven concerning the role of MSCs as a paracrine factory in promoting angiogenesis (Caplan and Dennis, 2006). VEGF secretion is one of the paracrine activities, which helps in neovascularization and angiogenesis (Ge et al., 2018). It also acts as a supporting cell for the micro-vessels, and in particular studies, it has been shown to act as pericytes that stabilize the micro-sized vessels and capillaries (Gerhardt and Betsholtz, 2003). Choi et al. (2022) showed that co-culture of MSCs and glomerulus endothelial cells promoted endothelialization at an optimized perfusion flow rate of 1 mL/min in a whole renal ECM scaffold. It was found that the flow rate exhibited injury response on the primary cell culture; however, co-culture of rat glomerulus endothelial cells (rGECs) and rat bone marrow mesenchymal stem cells (rBMMSCs) resolved this issue and resulted in increased proliferation and micro-vascularization in the glomerulus, reducing inflammation and cell death induced by the flow injury. This study showed that the use of MSCs, along with EPCs, could have a positive effect on improvising the vasculature development in a whole decellularized kidney matrix. MSCs are otherwise known to secrete a variety of ECM substrates such as collagen type I and III and fibronectin. These ECM components influence the fate of stem cell differentiation as well as angiogenesis (Lin et al., 2012). BMMSC-derived ECM can enhance the migration and proliferation potential of HUVECs in contrast to a tissue culture plate (Xu Y et al., 2014). Another study focusing on pre-vascularization of the whole decellularized matrix by BMMSCs or adipose tissue-derived mesenchymal stem cells (ADMSCs) showed efficient vascularization upon *in vivo* transplantation and functionality of the transplanted pancreatic islets in the decellularized liver seeded with either BMMSCs or ADMSCs. A 30-day follow-up showed improved blood glucose levels, while micro-CT results showed evidence of sprouting from the arteriovenous bundle inside the scaffold. Thus, MSCs could promote *in vivo* vasculature development in the decellularized matrix (Fathi et al., 2021). MSCs in whole-organ engineering could help achieve better outcomes, mainly due to their paracrine activity.

#### 4.1.4 Induced pluripotent stem cell-derived endothelial cells

With the first report of iPSC-derived endothelial cells (iPSC-ECs), the possibilities of creating a vascularized whole organ at a clinical scale has become more realistic. The current focus is on creating iPSCs, which are genomically stable and non-teratogenic, by using advanced technologies for inducing pluripotency for clinical use (Pa et al., 2015). Using iPSCs, researchers could derive both arterial and venous ECs (Rosa et al., 2019). Such differentiation prior to injection in a decellularized organ may, however, not lead to selective adhesion of arterial and venous ECs to the artery and vein, respectively. Therefore, strategies to differentiate arterial and venous cells must be delineated for complete coverage of the vascular bed, which perfuses the organ with oxygenated and deoxygenated blood. In a remarkable study by the Ott laboratory, iPSCs were used to derive ECs and vascular smooth muscle cells (vSMCs), which were transplanted into decellularized rat lungs in a two-step perfusion-based method for revascularization. Their method showed approximately 75% of endothelial coverage, which was further replicated in a human decellularized lung matrix. *In vitro* cultures for 3 days showed patent blood vessels and improved barrier function (Ren et al., 2015). Leuning et al. (2019) used iPSC-ECs for vascularization of a rat kidney. They developed a novel simultaneous arteriovenous delivery system for iPSC-ECs, showing the complete re-endothelialization of the kidney vasculature, including the glomerular and peritubular capillaries. They further replicated the study by recellularizing an entire human kidney with iPSC-ECs. This shows the utility of iPSC-ECs and possibly other related cell types which can possibly be used to re-engineering whole human organs, otherwise not possible by primary cells isolated from human tissues.

### 4.2 Growth factors

Angiogenic GFs have been the most commonly used biomolecules for inducing angiogenesis and neovasculogenesis in the decellularized whole-organ biomatrix for vascular reconstruction, the reason being the availability of abundant information about their kinetics and molecular and cellular characteristics. Molecules such as VEGF, platelet-derived growth factor (PDGF), basic fibroblast growth factor (bFGF), and bone morphogenetic protein 2 (BMP2) have been used in numerous studies accordingly. However, the primary reason for such a variable use of GFs makes it difficult for a biomedical tissue or organ engineer to select a defined cocktail of angiogenic mitogens in vascular reconstruction (Soker et al., 2000). There are studies where sequential release of a mixture of angiogenic GFs has been studied for angiogenesis. Freeman and Cohen (2009) showed that the vessel density and percentage of mature vessels in the sequential delivery of VEGF, PDGF-BB, and TGF-beta1 bound to alginate sulfate, with an affinity similar to that realized upon their binding to heparin, were three-fold greater in the triple factor-bound scaffolds than in the factor-adsorbed or untreated scaffolds. Moreover, vascularization within the triple factor-bound scaffolds appeared to be superior to that found in scaffolds delivering only bFGF. Such studies outline the importance of using multiple GFs for efficient and mature blood vessel development.

Another limitation in using GFs is their ability to interact with ECM components of the decellularized biomatrix. Such an interaction can significantly influence the availability of GFs to the nearby ECs and therefore affect or influence the proliferation as well as other angiogenic processes such as sprouting and tube formation. In the case of a neat biomaterial with no inherent GF-binding site, the kinetics of GFs can be controlled. However, in a decellularized biomatrix, control of the kinetics and GF-release behavior can be significantly compromised. Moreover, there is no defined decellularization protocol. Altogether, this results in the varied loss of ECM components spatiotemporally, eventually affecting the interaction and availability of GFs with the ECM components. To address this issue, first, the ECM components ought to be optimized by standardizing decellularization protocols, which can eventually help in the standardization and reproducibility of the undergoing vascular reconstruction processes (Matkar et al., 2017). One approach to address this issue is to develop combinatorial GF systems for inducing angiogenesis and vascularization in different decellularized organ matrices. It is important because the ECM glycosaminoglycans of different organs vary, and therefore, a more individualistic approach would be needed to reengineer blood vessels from a macro- to a micro-level. In an *in vitro* study (Davies et al., 2011), the ability of VEGF and PDGF-BB loaded on polyurethane-linked heparin scaffolds has been used to achieve *in vivo* vascularization. It was shown that after 1 and 3 months, covalent modification of the porous scaffold with heparin allowed for differential release of VEGF and PDGF-BB sub-cutaneously, which resulted in a rapid and sustained increase in scaffold vascularization. However, the decellularized whole-organ matrix appears to be more complex, and therefore, it would require further detailed studies related to the affinity, release, and spatiotemporal distribution of GFs regulating the angiogenesis and vascularization of mature blood vessels (Arenas-Herrera et al., 2013).

#### 4.2.1 Vascular endothelial growth factor

VEGF has been predominantly employed for the initiation of angiogenesis. This molecule is responsible for directional movement, growth, and proliferation of ECs. It also plays a role in the guided maturation of EPCs (Yang et al., 2012). VEGF acts through multiple signaling processes that contribute to both vascular repair and neovasculogenesis. For example, VEGF activates endothelial nitric oxide synthase, an enzyme that leads to nitric oxide production, which further stimulates EPC movement (Li et al., 2017). It has been demonstrated that ECs, when exposed to an insufficient amount of angiogenic growth factors, inhibit appropriate angiogenesis. These have been lately addressed by focused research on developing optimized carrier systems for stability maintenance and optimized delivery of GFs (Wagner et al., 2016). In this regard, VEGF, in combination with polyethyleneglycol (PEG), has been used to deliver VEGF in the decellularized whole liver matrix (DLS) to promote angiogenesis and patent blood vessel development. It was shown that the PEG–VEGF–DLS complex promoted better cell proliferation and differentiation of HUVECs than groups without PEG cross-linking. Moreover, the average density of blood vessels was higher in the PEG–VEGF–DLS group than in other groups at days 7, 14, and 21 after implantation *in vivo* (Zhang et al., 2022).

#### 4.2.2 Platelet-derive growth factor

PDGF is one of the most studied growth factor families. The molecule is mainly involved in several pathophysiological conditions. PDGF acts via paracrine signaling and is responsible for the proliferation, migration, and differentiation of a wide array of cells (Wang H. et al., 2016). These are primarily secreted in response to an injury by cells such as activated platelets, fibroblasts, epithelial cells, smooth muscle cells, and ECs (Bowen-Pope and Raines, 2011). The PDGF family consists of four genes, among which PDGF-C is mainly involved in vessel reconstruction via promoting differentiation of EPCs. ECs express PDGF receptors such as PDGFR-α and PDGFR-β. PDGF-C acts as a positive regulator of PDGFR-α and PDGFR-β, thus activating its downstream functions that involve increased vascularity and maturation of the vessel wall (Gilbertson et al., 2001). Although there exist no studies which specifically explore the effect of PDGF on vascularization of whole decellularized organs, the functional effect of PDGF has been extensively studied in acellular or decellularized tissues such as the aortic valve, blood vessel conduit, and urinary bladder. Interestingly, Zhou et al. (2012) showed that co-administration of PDGF-BB and VEGF in a bladder acellular matrix (BAM) enhances smooth muscle regeneration and vascularization in a rabbit model. The lyophilized BAM was hydrated with PDGF-BB and VEGF for 12 h and transplanted into a rabbit for SMC regeneration and vascularization. Although vascularization was significantly higher than that in the neat BAM control group, the release kinetics and loading remain to be optimized.

#### 4.2.3 Basic fibroblast growth factor

Studies have shown that bFGF works in conjugation with FGF to promote the migration of SMCs and fibroblasts into the graft to facilitate the formation of blood vessels. Among 22 different members of FGF, FGF2 has been the most investigated member for its *in vitro* and *in vivo* potential. FGF2 is a heparin-binding GF with multiple EC-binding motifs such as tyrosine kinase and heparin sulfate receptors that help induce various signal transduction pathways responsible for EC proliferation (Spinetti et al., 2001). FGF2 also helps in the activation of E2 (a target of the 17B-estradiol present in the endothelium) that leads to acceleration of re-endothelialization and an increase in circulating EPCs (Fontaine et al., 2006). bFGF increases the eNOS levels in HUVECs, leading to their proliferation and migration (Kitamura et al., 2014). FGF2 can stimulate ECs and also cause the release of MMP9 and MMP2, leading to the formation of capillary-like structures (Tarabo et al., 2002). These critical functions of bFGF make it an important GF for use in whole-organ tissue engineering. Although there exist no studies showing the effect of only bFGF on vascular induction and development in the whole decellularized organ, there is a huge repertoire of studies that have explored the role of bFGF in angiogenesis and vascularization in small tissues (artificially fabricated (Tabata and Ikada, 1999; Hori et al., 2003) and decellularized (Le et al., 2019; Lovett et al., 2009)).

#### 4.2.4 Bone morphogenetic protein 2

BMPs are multifunctional cytokines belonging to the TGF-β superfamily. BMP-2 is a crucial regulator of angiogenesis by facilitating the recruitment of EPCs through a concentration-dependent chemotaxis. During endothelialization, ECs dynamically undergo changes in the tip and stalk themselves. BMP-2 activates cells toward tip cell formation, while BMP-6 induces stalk cell formation, thus balancing EC plasticity during angiogenesis (Benn et al., 2017).

### 4.3 Small molecules

Small molecules are drugs with molecular weight less than 1 kDa and used as pharmacological agents with defined pharmacokinetic and pharmacodynamic properties for a target protein exhibiting a known biological function (Lu and Atala, 2014). Protein tyrosine phosphatase 1B (PTP1B) inhibitors are a class of molecules mainly studied for the treatment of type II diabetes. However, these inhibitor molecules regulate VEGF receptor 2 signaling and alternate p130/DCOK180/Rac1 pathways, all of which lead to endothelial cell motility contributing to the acceleration of re-endothelialization and formation of the functional vasculature (Wang Y. et al., 2016). The advantage of using small molecules in the vascular reconstruction of the decellularized organ biomatrix is their controlled pharmacokinetic and pharmacodynamic properties. However, it is not facile in the case of growth factors in a complex environment of the decellularized organ biomatrix. In addition to this, the low cost, ease of handling, and preparation make small molecules a preferred choice that must be explored in future studies on the vascular reconstruction of the decellularized organ biomatrix.

### 4.4 Vector-based gene expression

The re-establishment of a stable vascular endothelium is one of the critical points for whole-organ vascular reconstruction. It plays a crucial role in carrying out vascular functions and maintaining homeostasis, apart from supplying blood to tissues. Vector-based gene expression systems encoding various pro-angiogenic factors are an attractive alternative to the direct delivery of growth factors to establish a stable vascular endothelium. Due to the short half-life and stability issues associated with growth factors, gene-based alternatives provide growth factor secretion over a prolonged period to induce angiogenesis in a sustained manner. In addition, vector-based gene delivery provides more controlled expression of growth factors as an inducible promotor which generally controls it, thus eliminating the undesirable effects occurring due to overexpression/non-expression of growth factors.

Although vectors have not been utilized for re-endothelialization of the whole organ, the concept has been well established for tissue/graft vascularization. Vector-based gene delivery includes viral and non-viral vector systems. Mammalian vectors such as adenoviruses are the most widely used vectors for delivering genes as these display high efficiency of gene transfer. However, numerous reports suggest that these vectors can cause vascular inflammation, and sometimes, their random integration can also lead to hazardous outcomes (Kotterman et al., 2015). Contrary to these, insect-based viral vectors (e.g., baculoviruses) offer certain advantages over mammalian viruses, including their ability to replicate within mammalian cells and transduce non-dividing cells. Non-mammalian viral vectors also show lower cytotoxicity and inflammatory response than mammalian viral vectors (Paul et al., 2013). Non-viral systems for gene delivery involve either the transfer of a naked DNA plasmid encoding the gene of interest or the transfer of DNA through liposomal vesicles. Compared to viral vectors, these can carry much larger inserts and pose low biosafety issues relative to viral vectors (Sharif et al., 2012; Shah and Losordo, 2005). Furthermore, considering the extent of the vasculature that needs to be established for whole-organ reconstruction, a prolonged requirement of growth factors is necessary to avoid the formation of leaky/dysfunctional blood vessels. In this context, vector-based gene delivery can be used as a promising strategy for vascularization.

## 5 Strategies for re-endothelialization/vascular reconstruction in the DOB

**FIGURE 2:**
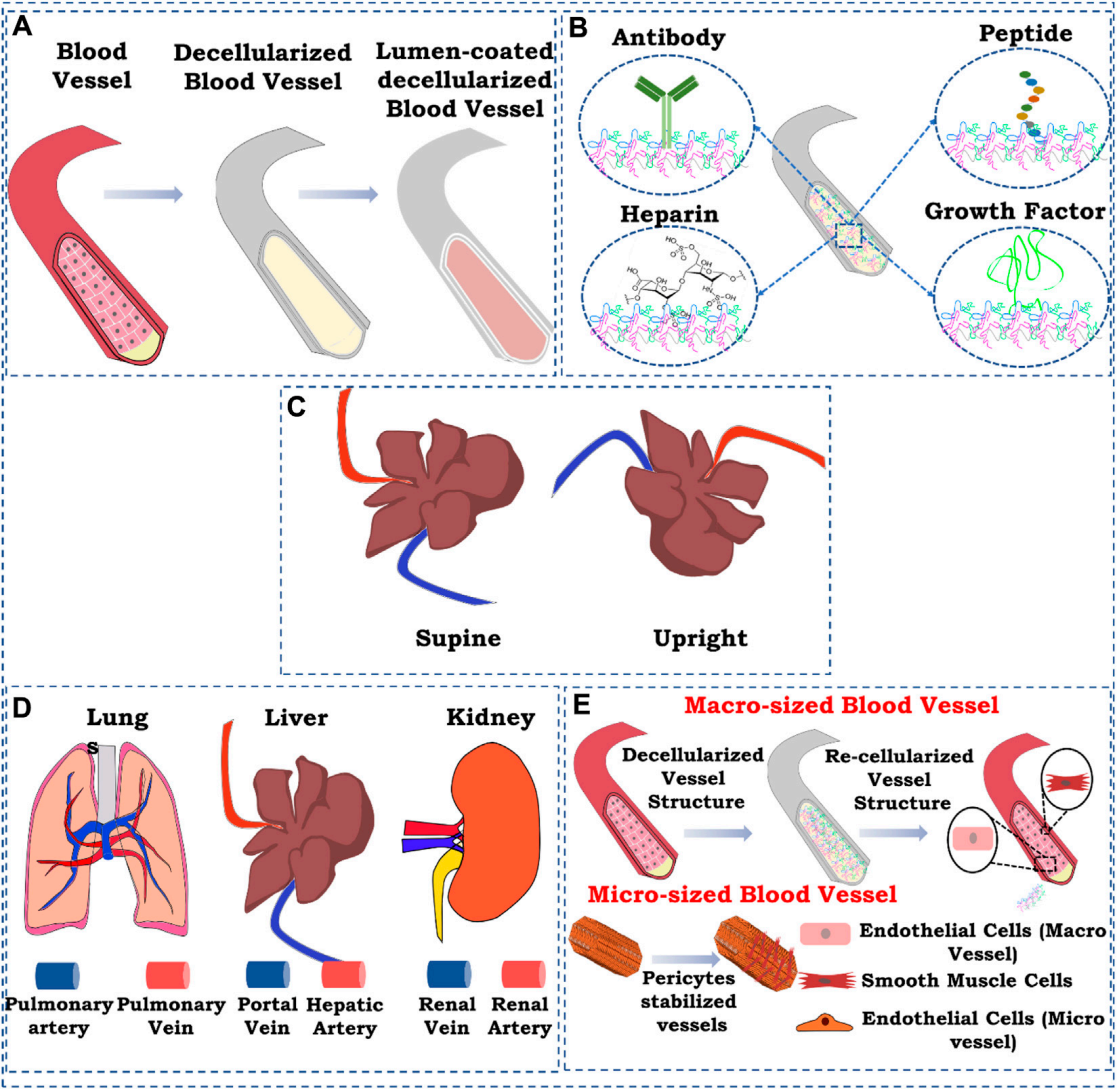
Schematic representation of strategies for re-endothelialization/vascular reconstruction in the whole decellularized organ. **(A)** Lumen coating of blood vessel structures; **(B)** immobilization, **(i)** antibody immobilization, **(ii)** heparin immobilization, **(iii)** peptide immobilization, and **(iv)** growth factor immobilization; **(C)** position of graft while seeding endothelial cells; **(D)** route of cell seeding; **(E)** re-endothelialization with supporting cells. The figures were prepared using Inkscape open-source vector graphics editor software, United States

Recently, several approaches have been explored to improve the re-endothelialization of patent blood vessels in the decellularized organ biomatrix, which are shown in Figure 2. This section aims to identify and highlight the approaches and delineates what innovation could be carried out in the future to achieve the eventual goal of vascular reconstruction.

### 5.1 Lumen coating of blood vessel structures

One of the most commonly used ways of improving re-endothelialization and vascular reconstruction is coating the lumen of the patent vessel structure in the decellularized organ biomatrix. This can provide a more suitable microenvironment in the lumen of the blood vessel structure for the endothelial cells to attach, proliferate, and attain morphology.

**FIGURE 3:**
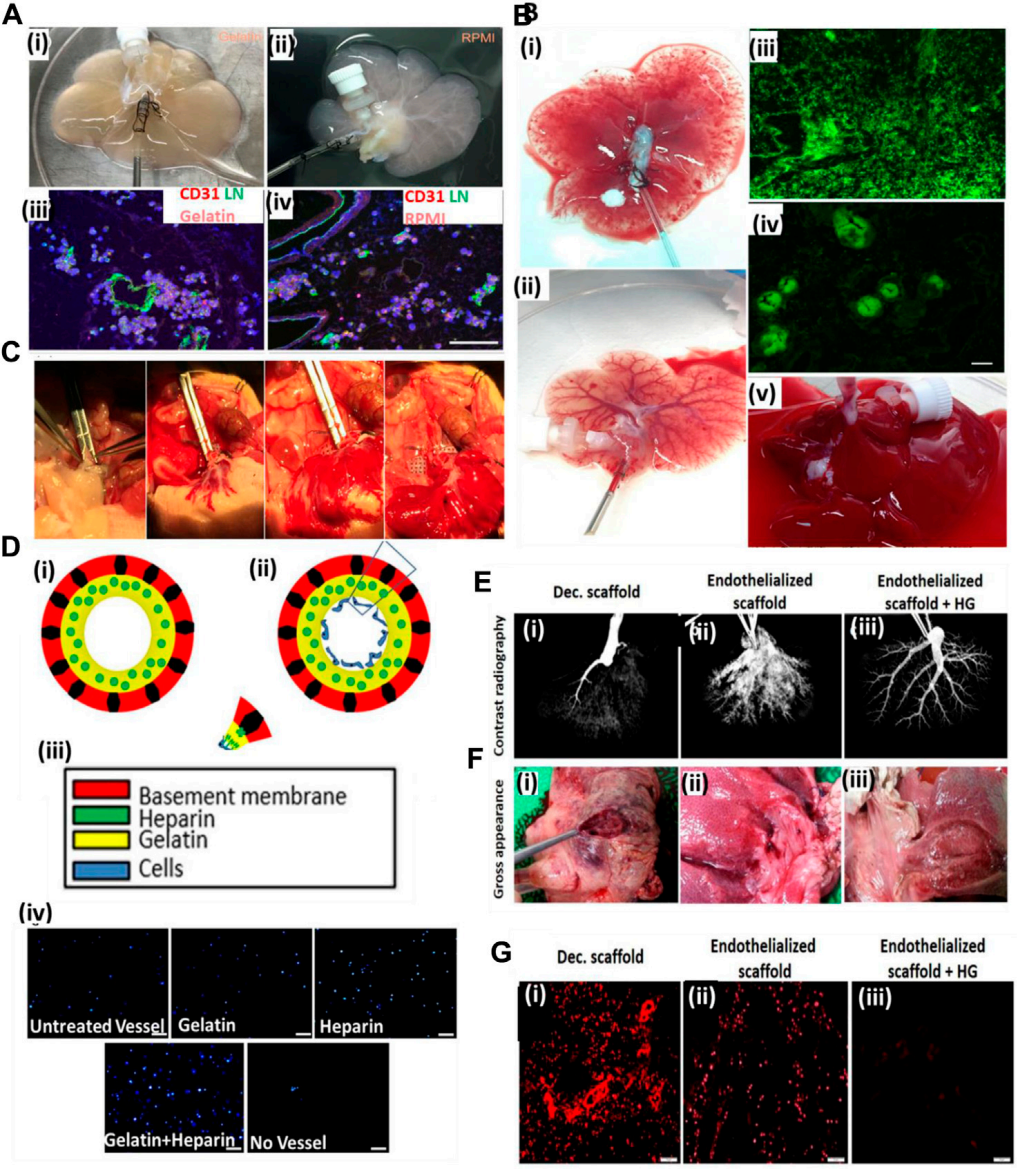
**(A)** Gelatin hydrogels and RPMI medium-based endothelial cell perfusion. **(i)** Appearance of the decellularized liver scaffold upon perfusion with gelatin hydrogel after 1 h of perfusion showed organ color visual appearance compared to **(ii)** RPMI medium-based perfusion. **(iii)** The gelatin hydrogel-perfused decellularized scaffold post-infusion with the endothelial–gelatin hydrogel complex showed formation of more and larger high-density endothelial cell aggregates compared to **(iv)** RPMI medium-based perfusion in the decellularized scaffold. **(B)** Studies to examine *ex vivo* blood perfusion of liver scaffolds. **(i)** Perfusion of the decellularized scaffold with blood showed exudation compared to **(ii)**, better retention of the blood, and improved vascular perfusion in the gelatin-based re-endothelialized liver scaffold exhibiting a clearly perfused vascular network of the liver scaffold. **(iii)** Infusion with GFP-positive blood showed extensive leakage or exudation in the extravascular spaces of the non-coated decellularized scaffold compared to **(iv)** gelatin-based re-endothelialized scaffold where GFP-positive blood was better retained within the vasculature of the re-endothelialized scaffold. **(C)** Heterotopic transplantation of the re-endothelialized liver scaffolds. The portal vein of the scaffold was able to sustain the arterial blood pressure as the initial blood flow into the transplanted scaffold commenced (ref. no. (A–B) (Meng et al., 2019)). **(D)** Hussein *et al.* showed the effect of the heparin–gelatin (HG) mixture on re-endothelialization of the decellularized liver. **(i)** Schematic representation of the overall study approach to improving endothelialization showing heparin–gelatin coating of the decellularized organ blood vessel luminal wall and endothelial cell attached on the luminal side after heparin–gelatin coating. **(iii)** Color coding representing various components of the re-endothelialized blood vessel. **(iv)** DAPI staining of the migrated cells under various coating conditions of the decellularized liver matrix. Scale bar = 10 μm. **(E)** *Ex vivo* blood perfusion of re-endothelialized porcine scaffolds. **(F)** Gross appearance showed that HG-precoated scaffolds were free from clots compared to uncoated re-endothelialized and decellularized scaffolds. **(G)** Immunostaining of decellularized scaffolds using anti-integrin αIIb antibodies demonstrated vigorous platelet adhesion and aggregation inside and around the vascular tree. Scale bar = 10 μm (ref. no. D–G (Hussein et al., 2016)).

Meng et al. (2019) used gelatin gel as a luminal coating material for improving the re-endothelialization of the decellularized liver. Coating of the decellularized liver improved the retaining of injected endothelial cells. As a result, endothelial cells lined the lumen of the vascular structure with increased vascular lumen coverage area and grew actively. Furthermore, the blood retention ability of the coated scaffold was improved, and *in vivo* studies using Doppler imaging confirmed the blood flow till day 8 post-transplantation (Figure 3A–C). Such results are significant because coating the lumen of the vascular tree in a decellularized scaffold can improve the perfusion and endothelial proliferation with the coverage area.

Similarly, Hussein et al. (2016) used a heparin–gelatin mixture to improve re-endothelialization in the porcine decellularized liver. Heparin was used as an anti-thrombotic mixture, and gelatin provided the matrix for endothelial attachment and migration. The re-endothelialized liver was cultured in a bioreactor for 10 days and was transplanted in a pig. Upon *in vivo* transplantation, the graft showed no thrombosis for 24 h and exhibited better HepG2 functions. Figure 3E shows the contrast radiography of HG-precoated scaffolds after 24 h as revealed by the uniform distribution of the contrast material within the blood vessels of the scaffold (3), whereas the contrast material could not penetrate the blood vessels of the uncoated scaffold margins after 16 h, which may have resulted from blood clots inside the vessels (2). The contrast material could not be perfused into the portal vein of the decellularized liver after 6 h because of blockage of the vasculature by blood clots (1). Figure 3G shows immunostaining of decellularized scaffolds using anti-integrin αIIb antibodies, demonstrated by a vigorous platelet adhesion and aggregation inside and around the vascular tree **(1)**, whereas the re-endothelialized scaffolds without HG show a relatively higher positive signal **(2)** than that of HG-precoated scaffolds **(3)**, indicating the necessity of efficient endothelialization to provide a non-thrombotic barrier and its ability to confine blood flow in the vascular spaces. This work was significant in laying the foundational structure in luminal coating and improving the decellularized organ extracellular matrix in vascular reconstructions.

### 5.2 Factor immobilization

Factors encompassing any biological molecules which improve the binding capability of endothelial cells in the decellularized organ matrix may promote efficient endothelialization. These biomolecules include endothelial cell-specific antibodies that promote endothelial cell attachment, peptide molecules that bind specifically to endothelial cells, growth factors that induce endothelialization by promoting the growth of endothelial cells, and, finally, biomacromolecules, such as heparin, that improve endothelialization in scaffold and tissue-engineered grafts.

#### 5.2.1 Antibody immobilization

**FIGURE 4:**
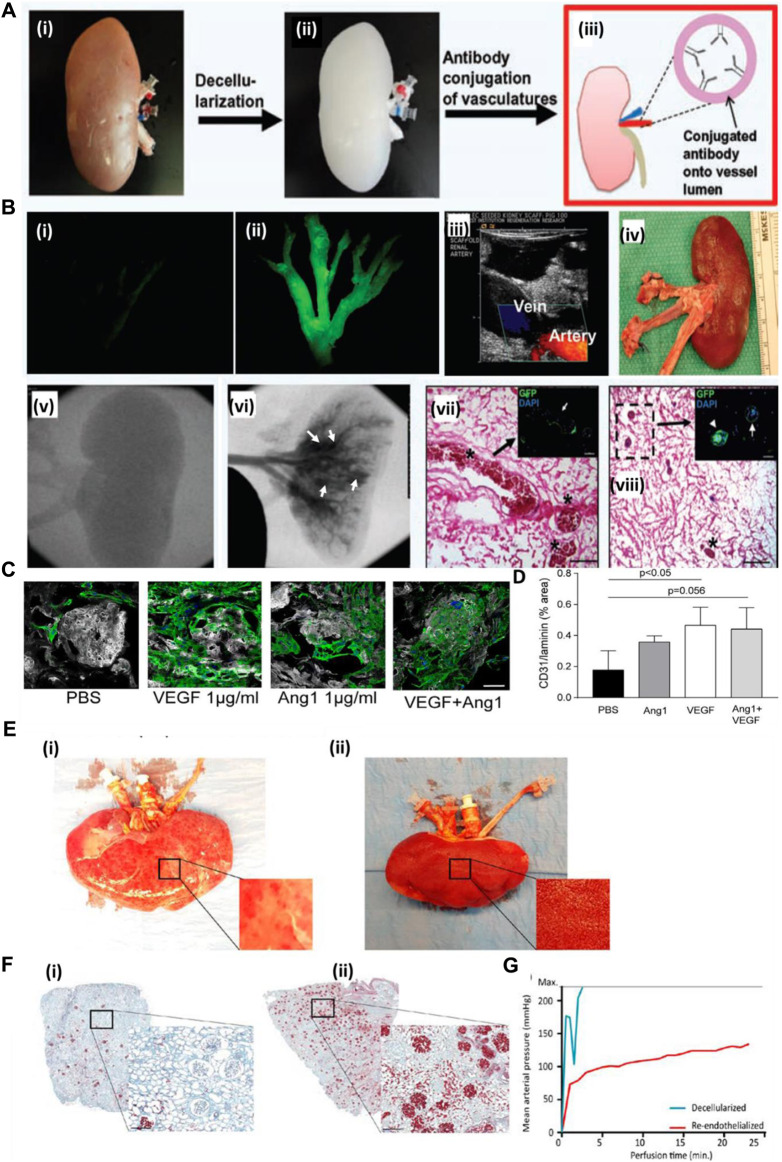
**(A)** Schematic representation of the antibody conjugation strategy for re-endothelialization of the decellularized kidney organ. Whole kidney **(i)** is decellularized **(ii)**, followed by conjugation of the antibody into the luminal side of blood vessels **(iii)**. **(B)** Decellularized kidney without GFP-positive-based re-endothelialization **(i)**, whereas GFP-positive endothelial cell-based re-endothelialization showed the vascular tree with proper endothelialization of the decellularized kidney vascular network **(ii)** (Ref. no. (A–B) (Ko et al., 2014)). **(C)** Scalability of hiPS‐EC culture and ability to adhere to growth factor‐loaded scaffolds. **(D)** Quantification of hiPS‐EC adherence after VEGF, Ang‐1, and combined loading. Ang‐1, angiopoietin 1; PBS, phosphate‐buffered saline; hiPS‐ECs, human-inducible pluripotent stem cell-derived endothelial cells; VEGF, vascular endothelial growth factor. Scale bar: 50 μm. **(E)** Human kidney re-endothelialization with hiPS‐ECs. **(F) (i)** Microscopic picture of Movat’s pentachrome staining of a decellularized human kidney cortex scaffold perfused with whole blood shows only a few perfused areas. Because of low pressure and massive leakage (due to the lack of endothelial cells), only a few red blood cells reached the glomeruli. **(ii)** When the human re-endothelialized kidney scaffold was perfused with whole blood, high coverage of blood in the cortex was observed (shown in red), showing that perfusion of blood through the entire kidney was possible. **(G)** Whole-blood perfusion of the decellularized human kidney scaffold was stopped within 5 min because of extremely high perfusion pressures, whereas the re-endothelialized kidney could be perfused for more than 20 min. Scale bar B and C: 500 μm; scale bar D, F, and H: 50 μm; scale bar E: 25 μm. White arrowheads: CD31 Dynabeads; black/white arrowhead: proliferating cell. Scale bar M and N: 200 μm. hiPS‐ECs, human-inducible pluripotent stem cell-derived endothelial cells; ns, nonsignificant (ref. no. (C–F) (Leuning et al., 2019)).

A decellularized kidney scaffold was re-endothelialized using the CD31 antibody conjugated within the graft. Upon antibody immobilization, endothelial cells were seeded and cultured in a bioreactor. The re-endothelialized kidney graft upon transplantation in a pig for 4 h showed patency of the vessel tree without extravasation of blood cells into the parenchyma of the kidney graft. This result showed that such a strategy of improving the endothelialization of a vascular tree with an endothelial-specific CD31 antibody improves the vessel coverage area and, eventually, the perfusion functions of the graft with clot formation and leakage (Figure 4A). Figure 4B shows the decellularized kidney without GFP-positive-based re-endothelialization **(i)**, whereas GFP-positive endothelial cell-based re-endothelialization showed the vascular tree with proper endothelialization of the decellularized kidney vascular network **(ii)**. An MS1-seeded kidney scaffold was implanted into pigs for 2 h (**iii)**. Ultrasound imaging at 1 h after implantation showed the artery and vein of the host blood perfusion of the re-endothelialized kidney organ **(iv)**. Gross appearance of the explanted scaffold post-implantation. Angiogram results of **(v)** before and **(vi)** after injection of the contrast agent demonstrated severe blood clots (asterisks in vii), as confirmed by histological examination **(vii, viii)**. MS1 detachment (arrows) and cell clogging (arrowheads) are observed in the images of boxes in g and h. The renal artery (RA), renal vein (RV), and inferior vena cava (IVC) are shown in the panels (Ko et al., 2014). Thus, such a strategy underlines the need to improve the vascular reconstruction using approaches that might improve the luminal coating of the vascular tree and blood perfusion.

#### 5.2.2 Growth factor immobilization

Growth factor immobilization has been explored for inducing angiogenesis in a tissue-engineered construct. However, the use of growth factors in the decellularized organ matrix is minimal. Leuning et al. (2019) attempted to recreate the vasculature of the kidney both at vessel and glomerular capillary levels. The authors loaded the scaffold with varying concentrations of VEGF in the decellularized scaffold. They studied iPSC-derived endothelial cell behavior and capillary formation both at the macro- and micro-levels. They observed that with an increase in VEGF concentration, attachment and survival of endothelial cells increased. Additionally, the rat decellularized kidney was re-endothelialized using VEGF at a concentration of 1,000 ng/mL, which helped increase endothelialization, adherence, and survival, based on *in vitro* studies. Figure 4C (i) shows representative images of hiPS‐EC adherence and survival after VEGF, Ang‐1, or combined loading of the scaffold. The same method was followed for re-endothelialization of a decellularized human kidney. Upon re-endothelialization, it was perfused with human blood *ex vivo* and observed that the re-endothelialized organ had much efficient blood detected by the presence of blood cells compared to the non-re-endothelialized decellularized organ, where clogging occurred within 5 min of blood perfusion. Figure 4E shows human kidney re-endothelialization with hiPS‐ECs: (i) decellularized human kidney perfused with whole blood showing only some “patchy” perfused areas; (ii) re-endothelialized human kidney scaffold perfused with whole blood showing diffused blood coverage over the whole kidney (Leuning et al., 2019). Therefore, this type of strategy must be explored to create blood vessels that are stable and functionally perfusable.

#### 5.2.3 Peptide immobilization

**FIGURE 5:**
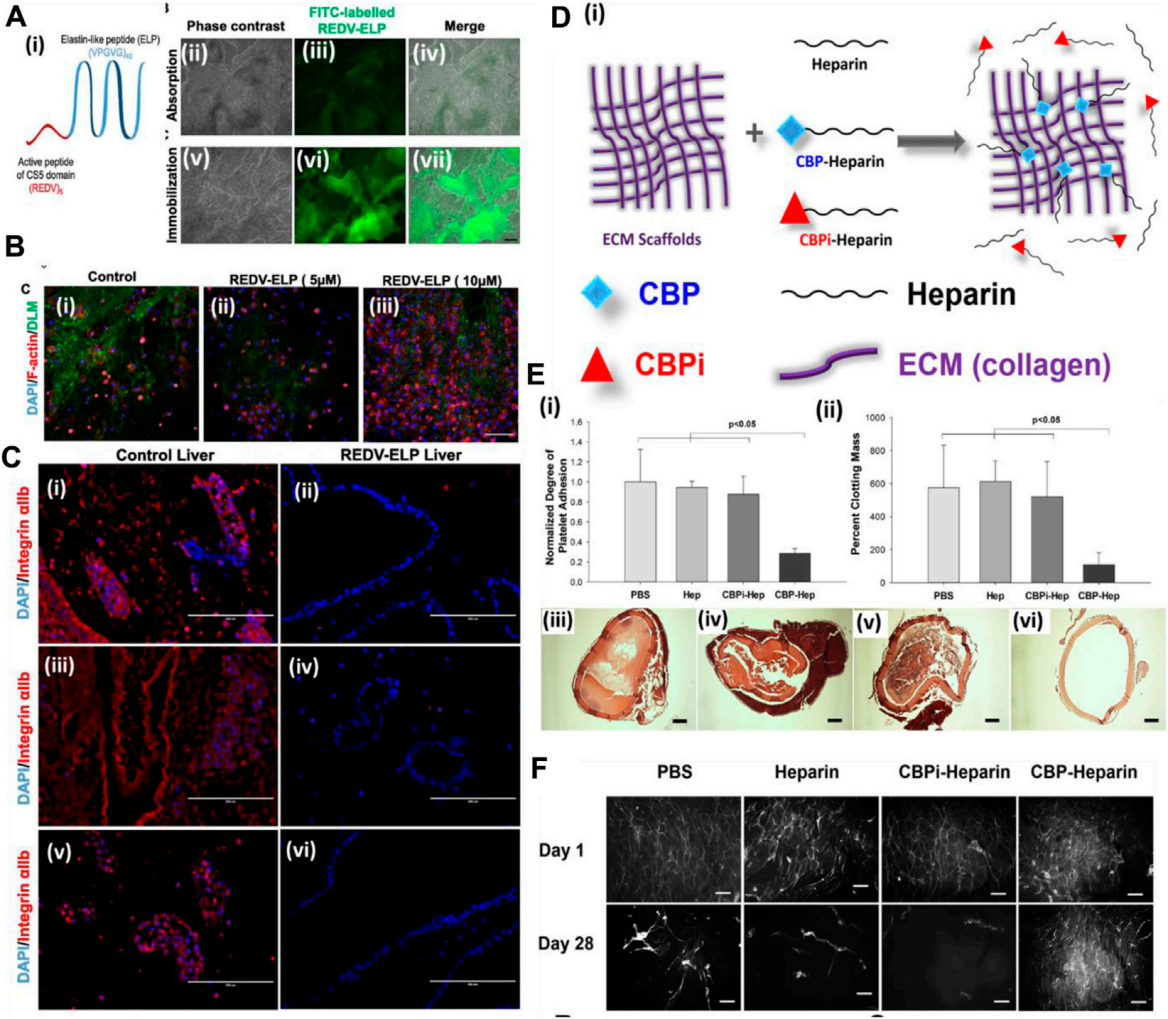
**(A) (i)** Schematic of the REDV–ELP fusion protein. **(ii–vii)** Micrographs of DLM discs conjugated with FITC-labeled REDV–ELP. Immobilization of the FITC-labeled ELP on discs was performed by absorption **(ii–iv)** or by covalent binding **(v–vii)** using EDC/NHS coupling chemistry and fluorescence visualized under a microscope. Scale bar = 500 μm. **(B)** Microscopic analysis of cell-binding properties of REDV–ELP-conjugated DLM F-actin staining for their morphology. **(i)** Non-conjugated DLM and **(ii)** DLM conjugated with 5 or **(iii)** 10 μM of the REDV–ELP fusion protein were allowed to attach with human EA.hy926 EC for 2 h. Staining for F-actin showed significantly better cell attachment on 10 μM of REDV–ELP fusion protein-conjugated DLM. Scale bar = 400 μm. **(C)** Immunostaining of re-endothelialized scaffolds using anti-integrin αIIb antibodies (red) and DAPI (blue). The absence of anti-integrin αIIb staining in REDV–ELP liver DLM **(ii, iv, vi)** showed non-induction of coagulation upon perfusion with blood compared to the presence of anti-integrin αIIb in the control **(i, iii, v)**. Scale bar = 200 μm (ref. no. (A–C) (Devalliere et al., 2018)). **(D)** Schematic representation of the ECM modification strategy. ECM is modified by CBP–heparin, but not CBPi–heparin or unfractionated heparin alone. **(E) (i)** Platelet adhesion to arterial ECM treated with PBS, heparin sodium, CBPi–heparin, or CBP–heparin (n = 3). CBP–heparin showed the lowest number of platelets binding compared to the ECM treated with PBS alone as a reference. **(ii)** Degree of clot formation by recalcified whole blood on arterial ECM with each separate modification showed CBP–heparin with lowest colt formation (n = 3). H&E images **(iii–vi)** show the degree of blood clot formation on ECM treated with **(iii)** PBS, **(iv)** heparin sodium, **(v)** CBPi–heparin, or **(vi)** CBP–heparin. Scale bar = 200 μm. CBP–heparin showed no clot/minimal clot formation compared to other groups. **(F)** Phalloidin staining of endothelial cells seeded on the lumen of the arterial ECM treated with PBS, heparin sodium, CBPi–heparin, or CBP–heparin at days 1 and 28 (scale bar = 100 μm) (ref. no. (D–F) (Jiang et al., 2016)).

A more cost-effective approach was explored for enhancing the endothelialization of the vascular structure of the decellularized organ extracellular matrix. Devalliere et al. (2018) used a peptide sequence immobilized on the vascular wall surface of a rat decellularized liver to improve endothelial cell attachment. In this work, the authors first synthesized a peptide by fusing cell-bound motif RGED with an elastin-like peptide (ELP) sequence that confers thermally triggered aggregation behavior of the fusion protein (Figure 5A). Then, the RGED–ELP was coupled to the vascular wall through portal vein infusion. Such conjugation improved cell attachment, spreading, and proliferation within the construct, leading to uniform endothelial lining (Figure 5B). This study also confirmed platelet adhesion and activation upon *ex vivo* blood perfusion (Figure 5C). Overall, this strategy exhibited a simple, cost-effective way of improving the endothelial lining of the vascular tree. Reduced platelet activation substantiates the hypothesis; however, no studies were performed to show the conjugation of such a small fragment peptide sequence in the parenchymal space. Such conjugation may improve capillary formation, promote better perfusion, and reduce platelet activation.

#### 5.2.4 Heparin immobilization

On similar lines, Jiang et al. (2016) showed the use of collagen-binding domain (CBD)-conjugated heparin in endothelialization and anti-thrombogenicity of the decellularized vessel graft. Heparin is known to bind with various growth factors as it is a naturally occurring macromolecule in the extracellular matrix of animals. Therefore, heparin use also promotes growth factor sequestration for more efficient endothelialization, apart from being an anti-thrombotic agent (Figures 5D–F). Wu et al. (2016) immobilized heparin in the porcine decellularized liver and loaded the heparinized decellularized liver with VEGF. They hypothesized that heparin is anti-thrombogenic and also harbors binding properties for growth factors such as VEGF. The prepared heparinized liver with sequestered VEGF showed significant blood vessel formation (*ex vivo* (CAM assay) and *in vivo* omental transplantation). In another study, the whole pancreas was decellularized and covalently bonded with heparin to check for thrombogenicity and perfusion capability. It was observed that immobilization of heparin increases the adhesion and proliferation of HUVECs, and upon *in vivo* transplantation of the heparinized pancreatic scaffold, the formation of a new blood vessel was observed. These studies indicate that the pro-angiogenic activity of the heparinized decellularized scaffold could be useful in reconstructing the vascular structure of the DOB (Xu et al., 2018). Such a strategy is quite helpful in controlling the angiogenic factor release behavior during angiogenesis. Too low or too high concentration/amount of angiogenic factor leads to improper vessel development. Therefore, GF sequestration and its release behavior would provide better control over endothelialization and micro-vessel formation.

### 5.3 Position of the graft while seeding endothelial cells

**FIGURE 6:**
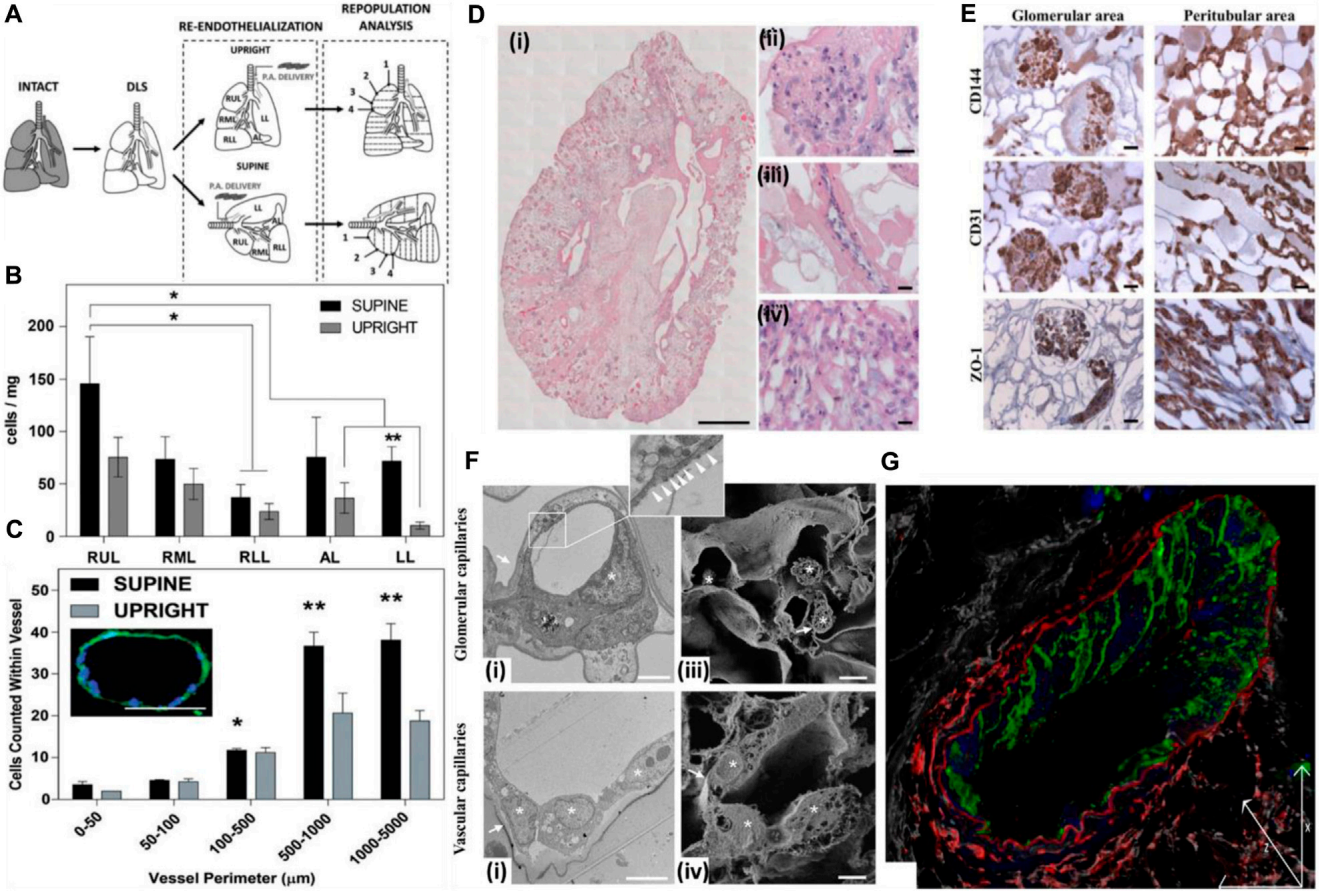
**(A)** Schematic for decellularization, re-cellularization, and analysis. The decellularized rat lungs were re-endothelialized in two anatomical positions to study the distribution of endothelial cells and effect of the position of the decellularized graft on re-cellularization via the pulmonary artery. Numbering represents sections of lobes from the apex to the base. SUPINE, lung seeded while lying in the supine position; UPRIGHT, lung seeded while held upright suspended by the trachea. **(B)** Distribution of ECs across the whole decellularized lung 1 h after seeding. The total cell distribution was based on estimates within each lobe and indicates that the supine position allowed greater and more even distribution of ECs seeded through the pulmonary artery. *The supine RUL cell count (cells/mg) was significantly higher than that for the RLL of the supine lung. **In the LL, the supine cell count (cells/mg) was significantly higher than the upright cell count according to t-tests. **(C)** EC counts within vessels of various sizes 1 h after seeding. Seeding ECs in the supine position allowed greater retention in the microvasculature than in the upright position. The image in inset represents a medium-sized vessel used for determining cell counts. This particular vessel had a perimeter of 410.02 μm with 18 nuclei counted within. Scale bar = 100 μm; magnification = ×20. (Ref. no. (Stabler et al., 2016)). **(D)** Reseeding of the kidney scaffold with iPSC-derived ECs delivered by the renal artery and vein. **(i)** Mosaic view of a transversal cross section of the repopulated kidney demonstrating a homogeneous distribution of iPSC-derived ECs into glomeruli and vascular structures. Scale bar: 1 cm. **(ii–iv)** Selected images showing iPSC-derived EC localization into the glomerulus (a’), vascular network (a”), and peritubular capillaries (a”’). Scale bar: 20 μm. **(E)** Characterization of the repopulated scaffold by CD144, CD31, and ZO-1 staining shows that iPSC-derived ECs maintain their phenotype in glomeruli and vascular structures. Scale bar: 20 μm. **(F)** Characterization of iPSC-derived EC repopulation by electron microscopy and confocal analysis. **(i)** Transmission electron microscopy image of glomerular capillaries showing repopulation by human iPSC-derived ECs, with the corresponding high-magnification inset showing a fenestrated endothelium indicated by white arrowheads. Scale bar: 2 μm; **(ii)** TEM analysis of small arteriole ultrastructures showing cell-to-cell contact between the endothelial cells. Scale bar: 5 μm; **(iii)** scanning electron microscopy image of glomerular capillaries. Scale bar: 2 μm; **(iv)** scanning electron microscopy image of vascular capillaries. Scale bar: 5 μm. In TEM and SEM images, capillary basement membranes and vessel walls are indicated by arrows, and endothelial cells are indicated by asterisks; **(G)** Z-sectioning and 3D reconstruction show a continuous layer of CD144^+^ ECs (green) in the vessel wall labeled with an elastin antibody (red). LCA lectin (white) staining shows renal structures, and DAPI (blue) staining shows nuclei. Scale bar: 25 μm (ref. no. (Ciampi et al., 2019)).

The position of the graft while seeding endothelial cells also affects the seeding efficiency, endothelial coverage, and re-endothelialization efficiency of the decellularized graft (Stabler et al., 2015). Gravity-assisted seeding of endothelial cells performed through the pulmonary vein (PV), and pulmonary artery (PA) of the decellularized liver showed a more homogenous distribution and better endothelial coverage than seeding through PV (Scarritt et al., 2018). Following this, another study focused on the endothelialization of decellularized scaffolds based on scaffold position while seeding endothelial cells. The decellularized liver was placed in either a supine or upright position, and endothelial cells were seeded through the pulmonary artery. It was observed that endothelial cells seeded in the supine position exhibited more homogenous seeding and better cell retention at the apex region of all the lobes than those seeded in the upright position. In addition, in the supine position, cell retention in larger blood vessels (proximal 100–5,000 μm) was more than that in the upright position (Figures 6A–C) (Stabler et al., 2016).

Similarly, in a separate study, when cells were seeded through gravity-driven hydrostatic pressure in a decellularized lung scaffold placed in a supine position, it was observed that the cells distributed evenly with better cell survival and proliferation than those seeded in an upright position while being submerged in media (Ren et al., 2015). These independent studies highlight the importance of the supine position over upright in increasing the re-endothelialization of patent blood vessel structures and cellular distribution, growth, and proliferation. This approach can also be explored in other organs such as the heart, liver, and pancreas. However, the anatomical location, structure, and position are different and affect overall seeding efficiency.

### 5.4 Route of cell seeding

The route of cell seeding is another aspect that must be optimized for each decellularized organ. The selection of route for the reseeded scaffold for both re-endothelialization and overall parenchymal cell seeding is very critical in homogenous cell distribution in general and endothelial cell lining and distribution in particular. Matthew et al*.* re-endothelialized a decellularized rat heart and improved the vascular reconstruction and decreased the thrombogenicity of the decellularized heart. They introduced rat aortic endothelial cells (RAECs) through three different approaches using three different routes: through retrograde aortic infusion, through brachiocephalic artery infusion, and through a combination of both inferior vena cava plus brachiocephalic artery infusion. It was observed that re-endothelialization using the IVC + BA route resulted in a redistribution of cells in the whole-heart scaffold. Introduction of RAECs through IVC and BA increased cellularity of the scaffold and resulted in higher endothelial lining of blood vessels. Re-endothelialization using the IVC + BA route significantly decreased *in vitro* and *in vivo* thrombogenicity of the re-endothelialized organ. It was also observed that re-endothelialization improved contractility of a left ventricular recellularized construct. Thus, vessel re-endothelialization through IVS + BA offers a superior vascularized scaffold with higher performance in whole-organ re-cellularization and *in vivo* vascular performance (Robertson et al., 2014).

In another study, iPSC-derived endothelial cells, when injected through the renal artery and vein of the decellularized rat kidney, resulted in a uniform distribution of cells in all the vascular compartments, from glomerular capillaries, peritubular capillaries, and small vessels. Endothelial cells showed continuity in small vessels, and further fenestration was detected in glomerular capillaries but not in the vascular capillaries **(** Figures 6D–G **)**. This is important from the viewpoint that introducing endothelial cells through both routes can result in the development of a site-specific fenestrated vasculature and intact continuous vascular capillaries in the same acellular kidney scaffold, thus underlining the importance of method optimization for endothelial cell seeding through particular routes for homogeneous cell distribution and development of an anisotropic vasculature network in a site-specific manner (Ciampi et al., 2019).

Baptista et al. (2011) studied the revascularization strategy in the decellularized liver of ferrets by intruding endothelial cells through two different routes: through the vena cava and portal vein. It was observed that when endothelial cells were seeded through the portal vein, the cells localized in the periportal region, whereas when seeded through the vena cava, cells were localized in the pericentral area. This study highlighted the importance of assessing different routes and the mode of endothelial cell seeding in a decellularized matrix for vascular reconstruction, ensuring intact and stable micro-vessel and macro-vessel formation with homogeneous cells distributed across all the sub-anatomic locations of the decellularized scaffold.

These studies provide fundamental information for vascular reconstruction, depicting that one delivery route is insufficient, especially in organs such as the liver and kidney, which have stable and intact large-sized blood vessels at the macroscale and fenestrated vessel microscale.

### 5.5 Re-endothelialization with supporting cells

Development of a blood vessel network in the decellularized organ upon *in vivo* transplantation exhibits several problems, such as fluid accumulation, blood clotting, and immune system reactions, such as macrophage accumulation. One of the reasons for such problems is impaired microvasculature development, which is unstable and leaky in nature (Carmeliet, 2003). One approach to address this issue is to mimic the native microvascular and micro-sized large blood vessel architecture and cellular composition which support the endothelial cells. These supporting cells are called mural cells, i.e., pericytes. Pericytes stabilize micro-vessels, and smooth muscle cells are found in the large blood vessel. Perivascular cells such as pericytes and fractional stromal cells form stabilized microvascular structures (Zuk et al., 2002). Such supporting mural cells stabilize the microvascular structure by secreting growth factors that regulate endothelial cell proliferation, sprouting, migration, and barrier function and permeability (Butler and Sefton, 2012). Doi et al. (2017) showed that decellularized lungs created the vasculature by seeding the organ with the adipose-derived stromal fraction, followed by seeding endothelial cells. ASC stabilized the endothelial network as observed by reduced vascular permeability and suppressed alveolar hemorrhage in an orthotopic rat model for up to 3 h after extubation.

## 6 Factors to be considered for vascular reconstruction in the DOB

**FIGURE 7:**
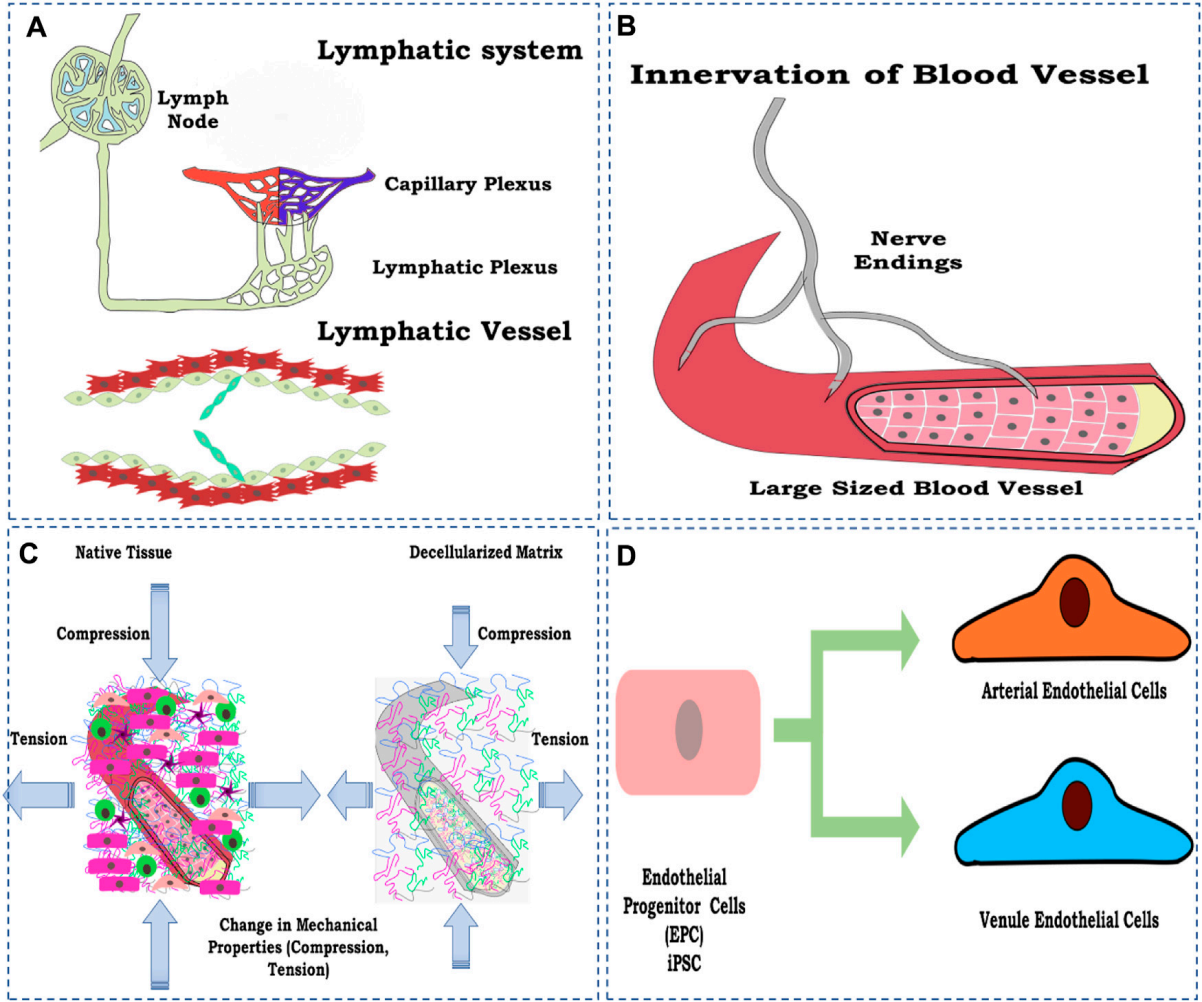
Schematic representation of the factors to be considered for vascular reconstruction in the whole organ, **(A)** lymphatic system development; **(B)** innervation of a blood vessel to control physiological response; **(C)** loss of mechanical property; and **(D)** maturing endothelial cells toward arterial and vein endothelial cell types. The figures were prepared using Inkscape open-source vector graphics editor software, United States.

Certain aspects have not been studied so far concerning whole-organ vascular reconstruction in general and parameters or factors that directly or indirectly control the vascular function and overall perfusion of the reconstructed whole artificial organ. Nevertheless, in general, some of the factors are discussed to emphasize the need for future studies focusing on the discussed parameters to be considered while designing the strategy of developing a fully functional perfusable whole organ in the laboratory (Figure 7).

### 6.1 Lymphatic blood vessel reconstruction

Many articles have been published since 2011 on the vascular reconstruction of the decellularized organ biomatrix. However, most studies focused on reconstructing blood vessels employing endothelial cells such as HUVECs, iPSC-derived endothelial cells, or E9 endothelial cell line. However, no reports focused on the reconstruction of lymphatic vessels using lymphatic endothelial cells.

The reconstruction of lymphatic vessels is significant due to their role in the transport and recirculation of interstitial fluid. Lymphatic fluid is vital for removing toxins from cellular metabolism and normal homeostatic function (Swartz, 2001; Breslin et al., 2019). Lymphatic fluid accumulates and flows into the left lymphatic vessels, which eventually drains into the right atrium of the heart and is recirculated back as blood (Szuba and Rockson, 1997). To date, almost no research has focused on the lymphatic reconstruction in the decellularized organ biomatrix and is, thus, one of the most critical aspects being neglected. Including lymphatics, the vessel, in principle, completes any given organ’s vascular perfusion system.

### 6.2 Innervation

Innervation is another process/strategy generally ignored during whole-organ reconstruction, especially in non-neural organs. One of the main reasons for this is the complexity involved in establishing neural networks within decellularized constructs. However, this poses one of the main hindrances in re-establishing a functional vasculature in re-engineered organs.

As we know, human organs are predominantly innervated, where blood vessels and nerve fibers reside in close contact with each other and thus modulate each other’s development and functionality. Blood vessels and nerves are also known to share common genetic pathways and respond to various common molecular signals. In addition, they also share anatomical similarities, and therefore, their growth is hugely interdependent (Das et al., 2020).

Reinert et al. (2014) established that VEGF, a key growth factor responsible for vascularization, plays a role in innervation and accounts for the post-natal growth of nerve fibers in pancreatic islets. Blood vessels allow for a sufficient supply of oxygen and other nutrients, an essential requirement for the growth of any tissue (here, nerve fibers). In turn, various neuropeptides secreted through neural networks influence angiogenesis. The calcitonin gene-related peptide 1 (CGRP-I) neuropeptide is known to promote endothelial cell proliferation and migration and enhance angiogenesis (Mapp et al., 2012). Endothelial cells possess β-neural growth factor (NGF) receptors that, upon interaction with NGF, induce a signaling process, leading to the secretion of VEGF and subsequent angiogenesis, thus establishing a co-relation where vasculogenesis and innervation considered an interdependent phenomenon in early developmental stages as well as healing processes (Marrella et al., 2018).

When engineering whole organs, it becomes imperative to establish autonomic connections with existing human neural networks to ensure the successful integration of engineered organs within the body. One of the crucial aspects of innervation besides interdependent growth is to allow for a coordinated action to occur between different cell types within the organ. For example, innervation is essential for coordinated and controlled vasoconstriction/vasodilation, ensuring proper blood flow within the organ. When engineering organs such as the heart, innervation is necessary to pump blood, control the heart rate, etc. Therefore, a mere establishment of a vascular network for whole-organ engineering without innervation can compromise the clinical relevance of such organ transplants.

### 6.3 Maturing endothelial cells toward arterial and vein endothelial cell types

It has been well established that arterial and venous endothelial cells are functionally different with respect to location and physiology. The shear stress experienced by arterial and vein endothelial cells is different and therefore exhibits different responses to change in hemodynamics and pressure both at the local and global levels (Dela Paz and D’Amore, 2009; Hussein et al., 2015; Carmeliet, 2000; Risau and Flamme, 1995) (1,2). Several factors have been uncovered related to the controlled divergence of differentiation of endothelial progenitor cells toward arterial and venule endothelial cell types, such as genetic factors, modifications by epigenetic factors, surrounding tissue secretions, hemodynamics, and oxygen tension (Hirashima and Suda, 2006; Othman-Hassan et al., 2001). In the vascular reconstruction of the decellularized organ biomatrix, the most general approach has been to re-endothelialize with HUVECs. However, there has been no report of re-endothelializing both the artery and vein with arterial and venous endothelial cells simultaneously in the DOB.

### 6.4 Loss of mechanical property

The overall composition of an organ constitutes cellular components, extracellular fibrillar and non-fibrillar components, and other glycosaminoglycans. The decellularization process so far explores the use of at least one detergent (ionic or nonionic) to remove the cellular and nuclear components from the organ. However, during such a process, many extracellular components, such as collagen, elastin, and other major fibrillar proteins, which form a significant bulk of mechanical properties, are lost depending on factors such as the percentage of detergent solution used, time for decellularization, and the sequence of use of decellularization (Hillebrandt et al., 2019). However, there is no standardized method of a decellularization strategy that can be used for any given organ. Therefore, this leads us to elucidate the best decellularization protocol for each organ, which must be optimized to lose its components, ECM matrix proteins, and glycosaminoglycans. In this regard, Mattei et al. (2014) studied the effect of decellularization on the mechanical properties of the porcine liver. Although this study covered two decellularization techniques, namely, agitation and immersion, the eventual outcome was in line with that of other studies about the decrease in elastic property of the decellularized organ slices compared to native tissue. These changes were observed due to the loss of collagen and glycosaminoglycan. However, decellularized slices and hydrogels have been used to study their angiogenic potential. However, to date, the replication or extrapolation of the angiogenic behavior and vascular reconstruction focusing on the mechanical status of the whole decellularized scaffold has not been conducted. It requires equal attention as mechanical behavior is a direct function of the ECM components, and the loss of ECM components directly influences *in vitro* angiogenesis and endothelial cell behavior.

## 7 Conclusion and future perspectives

The development of a fully functional perfusable artificial organ is a farfetched goal. However, it is achievable provided we understand the basic mechanisms underlying how vasculature development takes place from small capillaries *in utero* to large vessels during the development of the organism. At the same time, advancement in material science and technologies to deliver cells to specific locations within a decellularized matrix would help achieve the task of site-specific localization of specific cells. Lastly, standardization of cell derivation using a safe and reliable stem cell source for differentiation into various parenchyma and non-parenchymal cells would make it easier to construct a genuinely transplantable artificial organ. In the recent future, we should focus on optimization and standardization of the decellularization process for the derivation of fully functional patent vascular structures and the methods to re-create the vasculature, as highlighted in this paper. We expect that, with time, more technologies would uncover, which would help in the vascular reconstruction of the decellularized organ. Vascular reconstruction in the decellularized organ matrix is the most feasible process for developing a functional large-scale organ. It requires a systematic approach to solve multi-sectoral limitations in realizing the goal of developing a truly transplantable organ from the decellularized matrix. Cell selection is also limited by concerns such as government regulations, lack of intellectual property rights, and costs. The US Food and Drug Administration (FDA) regulates autologous human cells extracted and administered in a nonhomologous site (such as bone marrow cells transplanted to the liver) under *ex vivo* conditions as “manipulated” cells. However, it is good that there are different possibilities. The optimal strategy may be identified when clinical uses are investigated.
